# Exploring Near-Infrared and Raman Spectroscopies for the Non-Destructive *In-Situ* Estimation of Sweetness in Half Watermelons

**DOI:** 10.3390/foods13233971

**Published:** 2024-12-09

**Authors:** Miguel Vega-Castellote, Dolores Pérez-Marín, Jens Petter Wold, Nils Kristian Afseth, María-Teresa Sánchez

**Affiliations:** 1 Department of Bromatology and Food Technology, University of Cordoba, Rabanales Campus, 14071 Córdoba, Spain; g32vecam@uco.es; 2 Department of Animal Production, University of Cordoba, Rabanales Campus, 14071 Córdoba, Spain; 3 Nofima Ås—Norwegian Institute of Food, Fisheries and Aquaculture Research, PB 210, N-1431 Ås, Norway; jens.petter.wold@nofima.no (J.P.W.); nils.kristian.afseth@nofima.no (N.K.A.)

**Keywords:** portable NIR sensor, Raman analyser, measurement of *in situ* sweetness, half fruits

## Abstract

Watermelons are in high demand for their juicy texture and sweetness, which is linked to their soluble solids content (SSC). Traditionally, watermelons have been sold as whole fruits. However, the decline in the mean size of households and the very large size of the fruits, together with high prices, mainly at the beginning of the season, mean that supermarkets now sell them as half fruits. For consumers, it is important to know in advance that the fruits that they are purchasing are of a high quality, based not only on external flesh colour but also on sweetness. Near-infrared spectroscopy (NIRS) and Raman spectroscopy were used for the *in situ* determination of SSC in half watermelons while simulating supermarket conditions. A handheld linear variable filter (LVF) device and an all-in-one (AIO) Process Raman analyser were used for the NIRS and Raman analysis, respectively. The excellent results obtained—including residual predictive deviation for prediction (RPD_p_) values of 3.06 and 2.90 for NIRS and Raman, respectively—showed the viability of NIRS and Raman spectroscopies for the prediction of sweetness in half watermelons.

## 1. Introduction

Watermelons [*Citrullus lanatus* (Thunb.)] accounted for 11% of global fruit production in 2020 [1]. Although this fruit is mainly composed of water, it is a major source of antioxidant compounds, such as carotenoids, and sugars [2]. Watermelons are commonly sold as half fruits in supermarkets given their large size. Assuring the watermelon maintains optimal internal quality (once the fruit has been cut in half) is crucial to meet consumers’ demands, attain commercial acceptability, and guarantee sales of this valuable fruit [3]. One of the main parameters used to assess a watermelon’s internal quality is its sweetness, which in turn is correlated with the soluble solids content (SSC) of the fruit flesh. Recent developments in instrumentation, software, and data analysis have permitted the development of new alternatives to traditional methods to quantify different physicochemical parameters in fruits [4]. In particular, vibrational spectroscopic methods are considered to be among the most promising techniques for quality and process analysis in the food industry [5], and a fast and non-destructive alternative to the standard refractometry analysis used to measure SSC in fruits. In addition to ensuring a watermelon’s internal quality meets consumers’ demands, vibrational spectroscopy methods could also provide an alternative for taking quick measurements of the sugar content in watermelons in breeding programs that seek to enhance some of the fruit’s characteristics, such as flavour [6].

Applications using near-infrared (NIR) spectroscopy for measuring internal quality in fruits have been developed over recent decades [7]. Different studies can be found that aim to measure the SSC in watermelons once the fruit has been cut. In a study developed by Flores et al. [8], the potential of NIR technology to quantify SSC in freshly cut watermelon belonging to two different seasons was assessed, using a laboratory diode array instrument (Perten DA-7000, Perten Instruments North America, Inc., Springfield, IL, USA) working in the 400–1700 nm range. These authors cut a 6 mm-thick slice from the equatorial region of the fruit from which the NIR and SSC reference data were taken. Likewise, Ibrahim et al. [9] quantified the SSC in different portions close to the middle zone and to the stem of the flesh of watermelons harvested in the 2020 season. The methodology followed by these authors consisted of homogenizing these watermelon portions using a blender and taking the spectral data and reference values from the portions using a laboratory diode array instrument (Perten DA-7200, Perten Instruments North America, Inc., Springfield, IL, USA) working in the 950–1650 nm range. In this study, the NIR data were acquired by fitting the homogenized watermelon sample in a dish to be analysed with the laboratory NIR instrument. Finally, Vega-Castellote et al. [10] used a portable NIR device (MicroNIR^TM^ Pro 1700, VIAVI Solutions, Inc., San Jose, CA, USA) working in the 908–1676 nm range to measure in situ the SSC in half watermelons. These authors proposed an innovative application by taking spectral data from the surface of the half fruit to simulate an analysis at the supermarket level, as well as a reference value from the squeezed juice from a whole quarter of the watermelon flesh. In all cases, these studies showed promising results in the attempt to use NIR technology as a suitable tool for the analysis of cut watermelons.

Raman spectroscopy has been effectively employed for quality evaluation in food products, including olive oil [11], salmon [12], and pork [13], with good results reported in all cases. Although the number of studies available in the literature focusing on internal quality determination with this technology is significantly lower as compared to those using NIR spectroscopy, Raman spectroscopy appears to provide a feasible, alternative vibrational spectroscopy technique [14,15] for the non-destructive assessment of a fruit’s quality. In this context, Monago-Maraña et al. [16] quantified the SSC and individual sugars, such as glucose, fructose, and sucrose, in intact apples and in peeled apples using Raman spectroscopy, reporting promising results. Furthermore, Andersen et al. [17] stated that Raman spectroscopy was a suitable technique for the chemical and sensory characterization (total soluble solids, fructose, glucose, and citric acid contents, sum of acids, etc.) of fresh strawberries. In watermelons, Raman spectroscopy has been employed for the investigation of certain constituents, such as carotenoids in the rind [18]. However, the quantification of SSC in the flesh of this fruit using Raman spectroscopy has not been reported by any author in the literature. Nevertheless, the recent development of new low-cost, versatile Raman instruments has made this technology a viable tool with great potential for fruit evaluation.

No previous studies have been published that simulated supermarket conditions (without destroying the sample when taking the NIR information) and where the SSC data were taken from exactly the same areas of the watermelon’s flesh in which the NIR spectral measurements were carried out, which is of great interest given the heterogenous distribution of the sugars in the flesh of this fruit [19]. In addition, no studies have been published aiming at the determination of SSC in the flesh of half watermelons using Raman spectroscopy. Consequently, the objective of this study was to evaluate the feasibility of these two vibrational spectroscopy techniques for the determination of SSC in watermelon flesh, first using an NIR spectroscopy portable device and then a Raman spectroscopy instrument featuring a wide area illumination standoff Raman probe. In addition, this study includes a few final reflections regarding the strengths, weaknesses, and future prospects of the use of these spectroscopic techniques to analyse SSC in watermelon flesh.

## 2. Material and Methods

### 2.1. Sampling

To perform the NIR measurements, a total of 68 fruits were analysed in the Animal Production laboratory (University of Cordoba, Spain), while for the Raman spectroscopy analysis, 16 watermelons were analysed in the Nofima (Norwegian Institute of Food, Fisheries and Aquaculture Research, Ås, Norway) spectroscopy laboratory.

### 2.2. Instrumentation, Experimental Methodology, and Spectral Data Collection

#### 2.2.1. NIR Spectra Acquisition

The NIR spectral data were acquired using the MicroNIR^TM^ Pro 1700 (VIAVI Solutions, Inc., San Jose, CA, USA), a portable instrument that features linear variable filter (LVF) technology and works in the 908–1676 nm range, taking data every 6.2 nm in reflectance mode, with a 227 mm^2^ optical window size. The integration time of the instrument was configured to 11 ms, with 200 scans acquired for each measurement. The instrument’s performance was assessed at 10 min intervals using both white and dark references. To take the NIR data, the watermelons were first cut in half along the equatorial axis. Three equidistant measurements were taken in a straight line between the rind of the fruit and the centre, with the sensor placed in contact with the fruit’s flesh in static mode of analysis(punctual readings without the instrument moving during the measurement). A total of 204 point measurements were taken from the 68 watermelons analysed.

#### 2.2.2. Raman Measurements

The Raman spectral data were taken in a dark room with minimal ambient light using a MarqMetrix all-in-one (AIO) Process Raman system (MarqMetrix Inc., Seattle, WA, USA) and a wide area illumination (D = 3 mm) Proximal Fiber BallProbe HV standoff Raman probe. This system features a 785 nm laser working at 450 mW power. The spectral range of the system is 100–3250 cm^−1^ (100.000–3076.92 nm).

A 20 mm-thick slice was cut from the central part of the watermelon at the peduncle–pistil axis. A total of 4 20 mm-wide portions (cuboids) of this slice were used to perform the Raman measurements. The first and second cuboids were taken from the peduncle and pistil, respectively, to the centre of the slice. The third and fourth cuboids were taken along a perpendicular line from the equator of the slice to the centre. The resulting cuboids were 20 × 20 × L mm, with ‘L’ dependent on the dimensions of the slice. The spectral information was acquired from six equidistant points on the cuboid from the outer part (close to the rind) to the centre, with the cuboids placed at a distance of 10 cm from the Raman probe. The integration time was set at 20 s, and three spectra were recorded for each sample, which were then averaged to obtain the mean spectrum per point. A total of 384 point measurements were taken from the 16 watermelons analysed, with 4 cuboids per fruit and 6 point measurements per cuboid.

### 2.3. Reference Analysis

To measure SSC using NIR spectroscopy, a 1 cm-deep section of the surface below the sensor of approximately 227 mm^2^ from which the spectral data were obtained, was squeezed and the SSC was determined from the juice obtained using a temperature-compensated WAY-1S Digital Abbe refractometer (Xi’an Yima Optoelec Co., Ltd., Shanghai, China). For the Raman assay, slices of approximately 3 mm were cut from exactly the same points from which the Raman data were obtained on the cuboids. These slices (20 × 20 × 3 mm) were manually squeezed onto a Petri dish using a spoon, and the extracted juice was measured using a handheld TS Meter-D digital refractometer (Reichert Inc., NY, USA).

### 2.4. Multivariate Analysis of the Spectral Data

The MATLAB software version R2019a (The Mathworks, Inc., MA, USA) was employed to perform the chemometric treatment of the data.

Following the selection of the optimal spectral range for analysis, a principal component analysis was conducted on the NIR data, which were previously pretreated using standard normal variate (SNV) and detrending (DT) for scatter correction [20], as well as the Savitzky–Golay (SG) (polynomial order 2, window size 3) first derivative treatment. The Mahalanobis global distance (GH) was calculated according to Walsh et al. [21] to determine the distance between each sample and the centre of the population. The samples that presented GH values above 4 were studied and deleted from the set if the removal was justified. After studying the potential spectral outlier samples, around 20% of the watermelons (13 out of the 68 fruits) were randomly selected to be included in the validation set. The remaining fruits were used to build the calibration set. The prediction models for the SSC parameter were developed using the partial least squares (PLS) regression method, and segmented cross-validation was used, leaving one watermelon out each time; i.e., 3 point measurements, which is an appropriate cross-validation approach when there are structures in the dataset [22]. The first and second Savitzky–Golay (SG) (polynomial order 2, window size 3) treatments were tested along with SNV and DT. The best models were selected by assessing the values obtained for the coefficient of determination for cross-validation (R^2^_cv_), the standard error of cross-validation (SECV), and the residual predictive deviation for cross-validation (RPD_cv_), calculated as the ratio of the standard deviation (SD) of the reference data for calibration to the SECV. The best model was assessed using those samples included in the validation set and following the Windham et al. [23] protocol. In addition, during the validation process, the spectral distances were studied in detail, setting a limit for the statistic Hotelling’s T^2^ calculated at 95% confidence level, especially for those samples that presented a value above 2.5 for the Student’s t statistic [24]. This statistic was calculated as the ratio of the residual value for a given sample to the SEP.

In the assay carried out with the Raman system, the optimum range of work in the Raman shift (cm^−1^) was selected by removing the extreme ends of the range givento the presence of unwanted spectral variations. Next, the spectra were pre-processed using Savitzky–Golay (SG) smoothing (polynomial order 2, window size 9), and the fluorescence background was removed following Lieber and Mahadevan-Jansen [25], i.e., using MATLAB in-house scripts by subtracting a polynomial fitted to the baseline of the raw data using a polynomial degree of 6. Finally, the spectra were normalized using SNV. The pretreated Raman spectra were then interpreted, and the main peaks were identified. A PCA was applied to evaluate the presence of potential outlier samples, following the same criteria used in the NIR assays. To build the calibration and validation sets for the prediction of the SSC (%), 3 watermelons were randomly taken from the set of 16 fruits to be included in the validation set. The rest of the fruits were included in the calibration set. The calibration models were developed using the PLS regression method, and segmented cross-validation was used, leaving one watermelon out each time, i.e., 24 point measurements. To validate the models, the samples included in the validation set were used. The statistics used to assess the cross-validation and the external validation of the models were the same as those used in the NIR assays.

The PLS regression coefficients were then studied. Given the large number of variables with which the Raman instrument worked, new models were developed using only the most important regions identified from the regression coefficients plot, with the same calibration and validation sets as in the assays carried out using the whole instrument’s range of work.

Finally, to compare the RPD_p_ values obtained in the different assays carried out using the Raman device—when using the whole range and using only the variables in the most important regions to predict the SSC according to the regression coefficients—Fisher’s F test [26] was used, for which *p* = 0.05.

## 3. Results and Discussion

### 3.1. Characterization of the NIR and Raman Spectra

For the NIR assays, the whole spectral range from 908 to 1676 nm was selected. The characterization of the NIR spectra taken from watermelon flesh analysed in static mode using the MicroNIR^TM^ Pro 1700 device has already been discussed in Vega-Castellote et al. [10], showing different water (around 970 and 1450 nm) and sugar absorption bands (around 1200 and 1440 nm).

**Figure 1:**
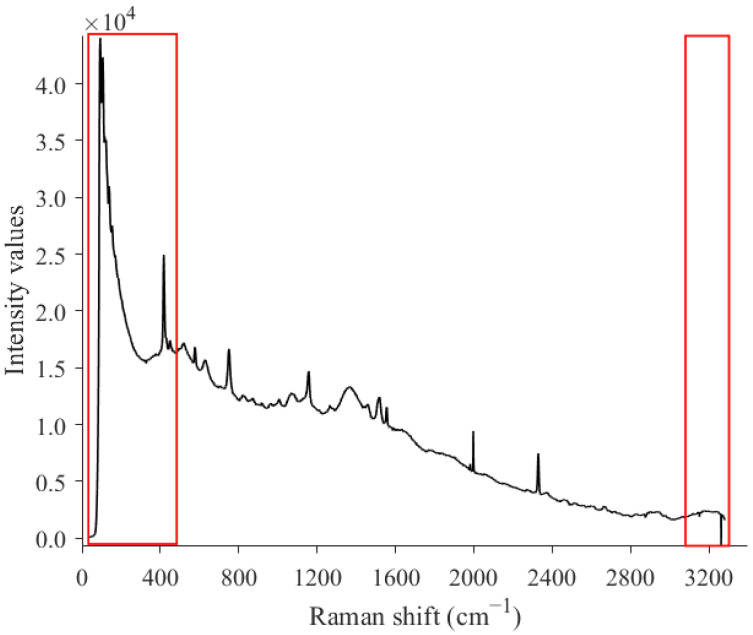
Raw Raman spectrum of a randomly selected sample. Red boxes indicate the spectral regions that were trimmed due to unwanted spectral variations. The 502–3052 cm^−1^ range was selected.

**Figure 2:**
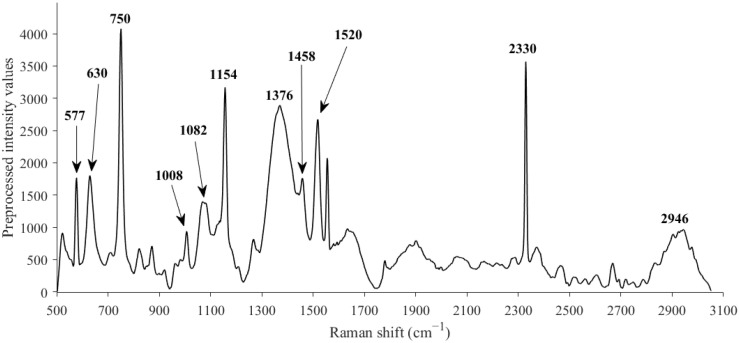
Main peaks of the mean pretreated Raman spectrum.

The Raman spectra were trimmed, and only the 502–3052 cm^−1^ range was selected due to unwanted spectral variations at the extreme ends of the range produced by the instrument in the acquisition of the spectral information (Figure 1). The mean spectrum of the samples acquired using the Raman instrument after baseline correction and smoothing pretreatments was then plotted (Figure 2). The Raman peaks from sugars and carotenoids are easily discernible, while in the NIR spectra, the absorption bands overlap, making the system complex and more difficult to interpret. In addition, the NIR spectra of watermelons are primarily influenced by the absorption of water, while Raman is almost insensitive to water. Two sapphire signals at 577 and 750 cm^−1^ can be observed in the Raman pretreated mean spectrum (Figure 2): these peaks appear as a result of the sapphire material present in the probe optics. Fructose peaks are shown at around 630 cm^−1^ [17], 1082 cm^−1^, and 1376 cm^−1^ [27]. In addition, the 1458 cm^−1^ peak could be associated with sugars, produced by the C-H bonds present in fructose, glucose, and sucrose [17]. Three important peaks related to the presence of carotenoids can be also seen at around 1008, 1154, and 1520 cm^−1^. The 1008 and 1154 cm^−1^ peaks may be linked to the stretching vibration of C-CH_3_ and C–C, while the 1520 cm^−1^ peak could be associated with the vibration of C=C [28]. A clear, sharp peak located at 2330 cm^−1^, possibly related to atmospheric N_2_ [29], can be also seen due to the relatively high exposure times used in our study. Finally, a peak at around 2946 cm^−1^ related to the presence of sugars, in particular fructose [27], can be also observed.

### 3.2. Exploratory Study of the Spectral Population and Characterization of the Calibration and Validation Sets

The PCA applied to the NIR dataset highlighted that only two of the samples presented GH values over four (GH = 4.20 and 6.07). The one showing a GH value of 6.07 also showed anomalies in the spectral curve. This sample had an SSC value of 11.35%, with the mean and standard deviation values of the whole set being 9.24% and 2.20%, respectively. This sample was removed from the NIR dataset. For the Raman dataset, a total of nine samples showed GH values over four (GH = 4.03, 4.38, 5.15, 5.53, 5.74, 5.86, 6.84, 8.36, and 8.82). Although none of them had an extreme SSC reference value, the samples with GH values of 5.53, 5.86, 6.84, 8.36, and 8.82 were removed from the Raman set, since anomalies associated with the spectrum acquisition process were found in their spectral curves.

After removing the spectral outliers, the NIR set comprised 203 samples, 164 of which were included in the calibration set and 39 in the validation set. Likewise, for the Raman assay, the 379 samples available after removing the spectral outliers were split into calibration and validation sets composed of 307 and 72 samples, respectively. The characterization of these sets is shown in Table 1. It is important to highlight that in both assays, the range of the validation set of samples was included in the calibration set. This is a crucial aspect to be considered since multivariate calibration enables us to obtain the best results when used as an interpolation method and not as an extrapolation method, according to Martens and Naes [30]. In addition, the calibration sets showed similar values to the validation sets for both the mean and the SD in the NIR and Raman assays.

### 3.3. Development of Models for the Prediction of SSC Using NIR and Raman Calibration Sets and PLS Regression

The best model for the NIR assay was obtained using the SNV, DT, and first derivative pretreated data and a total of 12 latent variables (LVs) in the PLS model (Table 2). These results reported that the PLS model showed excellent fit [31,32]. These results are of great interest for the non-destructive prediction of SSC in half watermelons on supermarket shelves. The results obtained were better than those obtained by Flores et al. [8] (RPD_cv_ = 2.50), who used a laboratory NIR instrument (400–1700 nm) to acquire spectral information from a 6 mm-thick slice, the size of the Petri dish, from which the reference data were also taken. Ibrahim et al. [9] used a visible (VIS)–NIR device working in the 475–1075 nm range and a similar sampling methodology to the one used in our study for the prediction of total soluble solids content (%), reporting an RPD_cv_ of 1.53. These authors also tested an NIR instrument operating in the 950–1650 nm range (cross-validation results were not given for the NIR system, only calibration results: R^2^_c_ = 0.94). Likewise, the results obtained in our study outperformed those obtained by Vega-Castellote et al. [10] (RPD_cv_ = 2.10). Nevertheless, a better match between the reference and NIR data could be expected in our study since the methodology followed by these authors aimed at estimating the sweetness of half fruits using the NIR spectral information by moving the sensor along the fleshy surface of a half watermelon and obtaining a mean SSC value per fruit.

**Figure 3:**
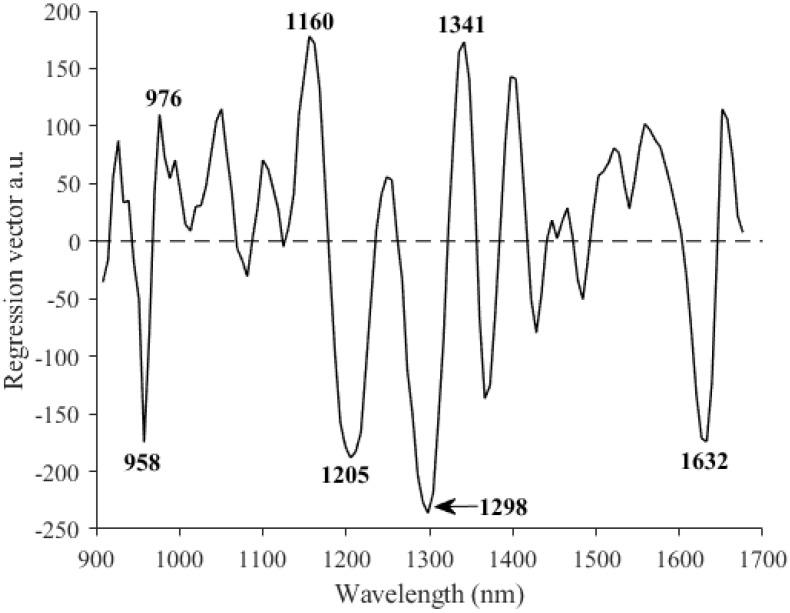
Regression coefficients for the best equation developed for the prediction of soluble solid content using the MicroNIR^TM^ Pro 1700.

The regression coefficient plot for the best model devised using the NIR instrument (Figure 3) showed a peak at around 958 nm, which could be associated with the water content [33]. The negative value of this regression coefficient could indicate that there is an inverse correlation between the water content of the fruit’s flesh and its SSC. As reported by Bianchi et al. [34], during the fruit ripening process, the SSC increases, causing a reduction in the fruit’s water content. The peak at around 976 nm could be related to the stretching of the O-H bond in the sugars [35]. The peak at 1160 nm could correspond to the C=O stretching [36], which in turn could be linked to the presence of aldehydes, which constitute one of the main flavour compounds in watermelons, with flavour being directly related to the fruit’s SSC [37,38]. A peak related to combination bands of C-H bonds present in carotenoids can be seen at 1341 nm [39]. The concentration of one of the main carotenoids in the watermelon flesh (lycopene) shows a similar evolution to the SSC during ripening, characterised by a marked increase in the first stages and a slight decrease once the fruit is overripe [2], which could explain the positive value of the aforementioned regression coefficient, which highlights the direct correlation between the two parameters.

After applying an SG smoothing filter to remove the background fluorescence and normalizing the data using SNV, the best results for the Raman assay were obtained with six LVs selected (Table 2). The lower number of LVs used here compared to the model developed using NIR data can be associated with the more complex nature of the NIR data compared to Raman [40,41]. As stated by Siesler [42], the NIR data are characterized by having wide, overlapped overtones and combination bands, in contrast to the Raman signals, which come from fundamental vibrations. The results obtained with the Raman instrument showed an excellent prediction capacity. Monago-Maraña et al. [16] also obtained good results for the prediction of SSC in peeled apples (SECV = 0.46%; R^2^_cv_ = 0.85), using one single LV in the PLS regression model. In addition, these authors reported excellent results for the prediction of individual sugars in peeled apples, such as glucose (SECV =1.9 mg/L; R^2^_cv_ = 0.91) and sucrose (SECV = 3.9 mg/L; R^2^_cv_ = 0.95), which opens up the possibility of predicting individual sugars in watermelons, as this is a key quality trait assessed in breeding programs of these fruits along with the total soluble solids content [43]. Similar results to the ones obtained in our study were reported by Andersen et al. [17] for the prediction of SSC in strawberries (SECV = 0.56%; R^2^_cv_ = 0.92) using PLS regression and five LVs. These authors also developed PLS models for the prediction of individual sugars, concluding that Raman spectroscopy would be a suitable tool for predicting these parameters.

**Figure 4:**
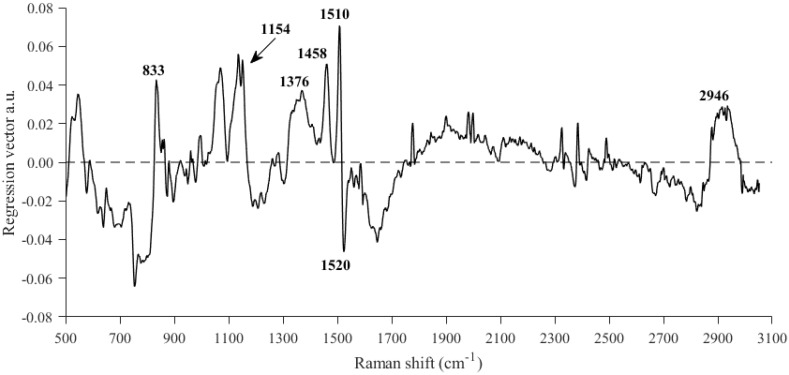
Regression coefficients for the best equation developed for the prediction of soluble solid content using the MarqMetrix AIO Raman system.

We then studied the PLS regression coefficients of the model developed using the Raman data. Most of the variables previously identified in the Raman spectra related to sugars and carotenoids (Figure 2) exercised a great influence in the model (Figure 4), such as the ones at 1154, 1376, 1458, and 2946 cm^−1^. In addition, according to Baranska et al. [44], the peak at 1510 cm^−1^ could be related to carotenoids and, in particular, to lycopene. These authors argued that this signal is asymmetric, with the 1520 cm^−1^ peak, which usually also assigned to carotenoids, related to β-carotene in this case. The positive and negative values of the peaks associated with lycopene and β-carotene, respectively (Figure 4), could indicate a direct, inverse correlation between the carotenoids and the SSC parameter.

As illustrated by the plot of the regression coefficients (Figure 4), the region between 1750 and 2800 cm^−1^ of the Raman shift showed little influence in the prediction of SSC in watermelon flesh. This could be expected since this region contains no relevant chemical information (Figure 2). The new PLS model was consequently carried out using only the 502–1750 cm^−1^ and 2800–3052 cm^−1^ regions of the Raman shift. After applying the same pretreatments used in the previous Raman model, the best results were obtained when selecting five LVs. Again, the results showed an excellent prediction capacity (Table 2).

### 3.4. Validation of the Models Developed Using the NIR and Raman Techniques

**Figure 5:**
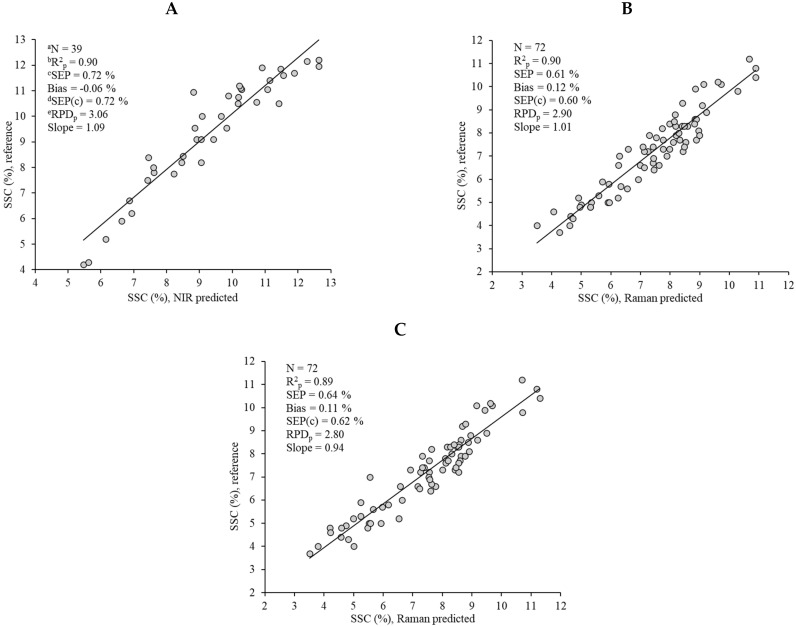
Reference vs. NIR predicted (**A**) and Raman predicted (**B**) values for the prediction of the soluble solid content using the full selected range, and reference vs Raman predicted values for the prediction of the soluble solid content using the MarqMetrix AIO in the 502–1750 and 2800–3052 cm^−1^ range (**C**). ^a^ N: number of samples; ^b^ R^2^_p_: coefficient of determination for the prediction; ^c^ SEP: standard error of prediction; ^d^ SEP(c): standard error of prediction bias corrected; ^e^ RPD_p_: residual predicted deviation for the prediction.

The best calibration model developed with the MicroNIR^TM^ Pro 1700, together with the ones carried out using the MarqMetrix AIO Raman device, were subsequently subjected to an external validation process. The model devised with the NIR instrument met all the requirements established in the Windham et al. [23] protocol, which highlighted the prediction capacity of the developed model. A strong correlation between the reference SSC (%) and NIR predicted values was obtained (Figure 5A) for that model, for which R^2^_p_ = 0.90 and RPD_p_ = 3.06, and with a slope of 1.09. After studying the residual predicted values for all the samples included in the validation set, none of them showed a Student’s t value above 2.5, and only one sample showed a Hotelling’s T^2^ value of 35.86 over the calculated limit of 23.53. The reference, predicted, and Student’s t values for that sample were 10%, 9.66%, and −0.47, respectively, and it was therefore not removed from the validation set. Given the results obtained, this model would enable us to predict SSC (%) with high precision and accuracy in cut watermelons and thus to determine non-destructively the quality of the half watermelons on supermarket shelves, to inform consumers about the sweetness level of the cut fruits, and therefore meet their demands.

Likewise, strong correlations between the reference and Raman SSC (%) predicted values were obtained using the full spectral range (Figure 5B) (R^2^_p_ = 0.90, RPD_p_ = 2.90 and a slope = 1.01). In this case, a total of five samples showed a Hotelling’s T^2^ value over the calculated limit of 13.00. These values ranged from 13.45 to 23.80. Nevertheless, the Student’s t value calculated for all of the samples in the validation set was under 2.5, and therefore, no samples were removed from this set.

In the validation process of the calibration model developed using only the most relevant parts of the Raman shift (Figure 5C), six samples were identified with a Hotelling’s T^2^ value over the calculated limit (Hotelling’s T^2^ limit = 11.38), with these values ranging from 11.45 to 27.63. No samples presented a Student’s t value over 2.5, and, consequently, no samples were removed from the validation set. This model yielded similar results (R^2^_p_ = 0.89, RPD_p_ = 2.80, with a slope of 0.94) to the one obtained using the whole Raman shift. No statistical differences (*p* > 0.05) were found between the RPD_p_ values of the two models (Figure 5B,C), which highlights the possibility of reducing the number of variables used to predict SSC (%) in watermelon flesh and thus the required computational time.

Although great prediction results were obtained using the Raman datasets, it is important discuss some aspects, as follows. First of all, the weaker signals of the Raman systems compared to the NIR ones resulted in longer exposure times for the former. The exposure time applied in this study could be a limitation for those applications in which great numbers of products need to be sampled in a short time. According to Lintvedt et al. [12], a reduction in exposure times can result in a lower signal-to-noise ratio, which, in turn, could be a limitation in the performance of the Raman prediction models. However, in those applications in which the speed of analysis is not a limitation, exposure time may not be as important. The sensitivity of the Raman technique to ambient light needs to also be taken into account. Analysis with this type of device needs to be performed in the darkness or under cold, blue light, the latter being of particular interest from an industrial operation point of view. To measure watermelons outdoors or in grocery stores, a black physical shield would have to be used to protect against ambient light. Finally, it should be mentioned that, in general, Raman instruments tend to be more expensive than NIR ones. Thus, developments in Raman technology need to focus on the optimization of exposure times and on producing Raman devices with more affordable prices. The development and improvement of Raman systems are still progressing rapidly, so we can expect the measurement time to decrease in the coming years.

## 4. Conclusions

The quantification of SSC in watermelon flesh was successfully determined using NIR spectroscopy (R^2^_p_ = 0.90 and RPD_p_ = 3.06). Given the heterogenous distribution of the sugars in the flesh of these fruits, the obtained results are of particular interest for the non-destructive prediction of SSC in half watermelons on supermarket shelves, since no previous study has been published following the methodology proposed in our study, where the SSC data were taken from exactly the same areas of the watermelon’s flesh in which the NIR spectral measurements were carried out (without destroying the sample when taking the NIR information).

Likewise, excellent results were obtained using Raman spectroscopy, demonstrating the possibility of using this technique for the non-destructive determination of SSC in the flesh of watermelons, which had not been proved before. In this case, when the model was developed using only the most relevant parts of the Raman shift, similar results (R^2^_p_ = 0.89, RPD_p_ = 2.80) were obtained compared to when the whole Raman shift was used (R^2^_p_ = 0.90, RPD_p_ = 2.90). In particular, the RPD_p_ values showed no significant differences (*p* > 0.05), which indicates that the number of variables used for the prediction of SSC (%) in watermelon flesh can be reduced, thus reducing the required computational time. Specific limitations were found in the Raman analysis, such as the technique’s sensitivity to ambient light—which could be solved by performing the analysis in darkness or under cold, blue lights—or the long exposure times required, for which we can expect a reduction in upcoming years given the fast and continuous development and improvement of the Raman systems. Nevertheless, it is important to conclude that the application of Raman technology for the prediction of individual sugar contents in watermelons could be of great interest in breeding programs for this fruit.

Although the number of fruits used in this study was limited, a great number of paired reference and spectra were used both for the NIR and Raman assays (204 and 384, respectively), allowing for great variability in terms of the SSC of the watermelon flesh. Nevertheless, as this can be considered a preliminary study for the assessment of NIR and Raman spectroscopies for the non-destructive *in situ* estimation of sweetness in half watermelons, further studies will focus on including a greater number of fruits in view of building a more robust model for routine analysis operations.

## Figures and Tables

**Table 1:** Characterization of the calibration and validation sets used for soluble solid content determination using the MicroNIR^TM^ Pro 1700 and the MarqMetrix AIO.

Instrument	Set	^a^ N	Range	Mean	^b^ SD	^c^ CV (%)
MicroNIR^TM^ Pro 1700	Calibration	164	3.80–14.15	9.21	2.21	24.00
	Validation	39	4.20–12.20	9.39	2.22	23.64
MarqMetrix AIO	Calibration	307	3.40–12.5	7.84	1.97	25.13
	Validation	72	3.70–11.20	7.20	1.79	24.86

^a^ N: number of samples; ^b^ SD: standard deviation; ^c^ CV: coefficient of variation.

**Table 2:** Best calibration models obtained for the prediction of soluble solids content (%) using the MicroNIR^TM^ Pro 1700 and the MarqMetrix AIO.

Instrument	^a^ N	Range	Mean	^b^ SD	^c^ LV	^d^ SECV	^e^ R^2^_cv_	^f^ RPD_cv_
MicroNIR^TM^ Pro 1700	155	3.80–13.30	9.14	2.16	12	0.56	0.93	3.86
MarqMetrix AIO (full spectral range)	294	3.40–11.80	7.80	1.94	6	0.60	0.91	3.24
MarqMetrix AIO (502–1750 and 2800–3052 cm^−1^ range)	293	3.40–11.80	7.80	1.94	5	0.63	0.90	3.07

^a^ N: number of samples; ^b^ SD: standard deviation; ^c^ LVs: latent variables; ^d^ SECV: standard error for cross-validation; ^e^ R^2^_cv_: coefficient of determination for cross-validation; ^f^ RPD_cv_: residual predictive deviation for cross-validation.

## Data Availability

The original contributions presented in the study are included in the article, further inquiries can be directed to the corresponding authors.
